# The Parent Tissue and the Blastema

**Published:** 1857-09

**Authors:** W. S. Bowen

**Affiliations:** New York


ART. II.—*The Parent Tissue and the Blastema.* By W. S. BOWEN, M.D., of New York.

THE following quotations, though seemingly disconnected, are united in this, that they turn upon the same question, viz.: the Chemical Constitution of the Blastema, and the influence of the parent tissue in the formative process.

Or, they may be grouped under the head of Phases of Science, as indicating an admixture of error or doubt.

Thus: It was a phase of science that Dr. Vogel presented when he observed* that "the only (solid) cyto-blastema which has yet been noticed, in relation to morbid products, is coagulated fibrine, in its amorphous condition, and permeated with water." "It is possible," he adds, "although it has not yet been observed, that other protein compounds—albumen, casein, or globulin—may act as cyto-blastemata."

Dr. Lehmann approached nearer to the truth, when referring to this paragraph, he remarked,† "A careful consideration of plastic exudations will, in itself, lead us to doubt the correctness of Vogel's views." "We cannot suppose Vogel intends to assert, that it is only the fibrine of the exudations which is converted into cells and fibres." "It still remains, however, for us to determine why cells and fibres are not formed from serous exudations; that is to say, from albuminous solutions, containing no fibrine."

"It would seem," Dr. Lehmann continues, "that the serous exudations possess no tendency to become organized" (? not) "in consequence of their never being pure plasma minus fibrine, but of their frequently containing less albumen, and, in all cases, more salts and extractive matters than the serum of the blood. Although we are unable to determine the manner in which the salts are able to arrest the metamorphosis of albumen into cells, we yet know that other metamorphoses of albumen, as for instance, putrefaction, are hindered or modified by the agency of these bodies."

This is a phase of science, but so remarkably near what we now suppose to be the truth, that it is absolutely involved in the expressions employed.

Suppose, for instance, Dr. Lehmann had said that each separate tissue and secretion is composed of definite proportions of its constituents, and that no substance, albuminous or otherwise, can form a blastema, for their development, which does not contain, for each, the necessary elements in proper form, it would then have been seen that the excess of salts had converted the albuminous solution into a chemical compound, incapable of

* Pathological Anatomy of the Human Body, by Julius Vogel, M.D., pp. 105–6: London, 1847.

† Physiological Chemistry, by Professor C. J. Lehmann, vol. i, pp. 347–8: Cavendish Society Edition.

receiving and exerting those influences which are brought into active exercise between the parent tissue and the blastema, when all the essential conditions of the formative law are present. This Dr. Lehmann expressed, but the expression was obscure.

It was a phase of science that presented no appearance of truth, and which led the profession into error, and confirmed erroneous practice, when Mr. Paget announced,* authoritatively, that "Few theories have been less founded than those which regarded scirrhous or medullary cancer, as in any sense the result or sequence of inflammation."

It would seem necessary to the full comprehension of a subject, though but little flattering to our vanity, that not only the general principles should be given, but illustrated, and that the practical application should be pointed out. In short, that nothing be left to intuition—nothing to induction.

Let us glance, in passing, at the relevant facts of science, known by their authors, when the above quotations were written.

Dr. Vogel knew that the egg contained no fibrine, and that from albumen and vitellin, carbonate of lime and phosphorus, the tissues of the bird had to be formed; and he had a comprehensive knowledge of the law of homologous formations. He knew that the law of analogous formations was derived from *it*, and that heterogeneous products ensued as a still more remote result of the modified conditions of the first-named law; for when speaking of the various forms of condensation of tissue, he remarks, "We shall attempt to work out, as far as possible, the general laws followed by pathological formations in their development. These laws are very closely allied with those which direct the development and formation of tissues in the normal state; indeed, in many cases no definite line can be drawn between normal and abnormal formations" (p. 99). "The cyto-blastema, on the one hand, and the pre-existing tissues on the other, are each factors influencing the formation of organized morbid products, and it is on their different properties that these epigeneses are dependent, both for their mode of formation and for their general characters."

By "properties" we are to understand, on the part of the blastema, its "quantity" and "quality," and as they refer to the parent tissue, its state of health, or "physiological type." "In proportion as the physiological properties of the parent tissues deviate from the normal type, so much the more heterogeneous will be the epigenesis. Thus in gangrenous parts, the exudation admits of no normal development, and the same is the case in parts in which the nerves have been divided. In structures which have been changed by chronic inflammation, or of which the physiological properties of the elementary parts differ, for any reason, from the normal type,

* Lectures on Surgical Pathology, by James Paget, F.R.S., vol. 2, p. 557. London, 1853.

pathological epigeneses are produced distinct from those that occur in healthy parts" (pp. 114–116). No error can be discovered here, except that noticed by Dr. Lehmann, relating to the constitution of the blastema, and it is more than probable, as Dr. Day suggests in his translation of the "Physiological Chemistry," that Vogel's remark applied exclusively to morbid products. This view, however, does not impair the force of Lehmann's criticism, fibrine not having been named as a transition substance, but as THE pabulum, and subject to metamorphosis.

Lehmann corrected Vogel: yet had he studied his author with more care he would, himself, have given us a fact where he presents but a phase of science. Had he given that attention to the quality of the blastema which Vogel indicated as essential, and had he in connection with this studied the constitution of the different tissues and secretions, he would have arrived at the knowledge of what elements were required in any given case to form an elaborate blastema. He knew that albumen was the potential basis of all the structures, and that, as for the nutrition of the whole body, albuminous solutions were insufficient without the inorganic principles, so for each separate tissue or secretion, some one or more of these would be required, in addition to albumen, to form its nutritive material.

The application of this knowledge was neglected, and the result was an approximation to the whole truth, whereas the complete theory might have been given unmingled with hypothesis.

So at the present day we see men questioning the significance of cells in the formative process. All that is required on the part of Mr. Huxley is to lay aside the microscope and resort to chemical investigation, and he will connect the various appearances of cells and fibres with certain formulæ; and throw a steady light over the whole subject of metamorphosis and development.

Mr. Paget was familiar with all the learning that distinguished Drs. Vogel and Lehmann—he had read the paragraph just quoted: "In proportion as the physiological properties of the parent tissue deviate from the normal type, so much the more heterogeneous will be the epigeneses"—yet he says that cancer can never result from inflammation.

I propose to arrange the conditions essential to formation in concise formulæ, in order to show the facility with which a purely physiological process may glide into morbid action, and at the same time I hope to demonstrate the reverse of Mr. Paget's position.

It is but just to this gentleman, with whom it is my misfortune to differ, that his arrangement of the conditions of nutrition should be placed prominently in view, in order that the reader may judge intelligently of the argument.

"Doubtless," Mr. Paget remarks, "the conditions necessary to the normal nutrition of parts are many, but the chief of them are these four:

"1. A right state and composition of the blood, or other nutritive material.

"2. A regular and not far distant supply of such blood.

"3. (At least in most cases), a certain influence of the nervous system.

"4. A natural state of the part to be maintained." Vol. I, p. 15.

By a right state of the blood, we are to understand a fluid capable of furnishing an appropriate blastema.

The following are the conditions of Dr. Vogel's law of analogous formations:

"The nature of the development of pathological epigeneses is dependent on

"1. The cytoblastema, the quantity, quality, and mode of its production. The more rapidly and abundantly it is secreted, and in proportion as the chemical composition (which, however, is certainly not accurately known), differs from the normal blood plasma, so does the influence of the surrounding histological elements decrease, and the formation proportionately deviate from the normal type. Thus, small quantities of exudation become easily organized, and simple hypertrophy usually consists in small exudations repeatedly occurring, after long intervals (weeks or months). We can draw no very definite limit between this process and that of healthy nutrition. Abundant and rapidly formed exudations are rarely organized, usually proceeding to suppuration. Exudations undergoing incipient putrefaction, as for instance, ichor, do not beeome organized, and where the composition of the blood (and, consequently, the exudation proceeding from it) differs considerably from the normal type, as is probably the case in typhus and scrophulosis, either no organization follows, or else it occurs very imperfectly.

"2. The nature of the development is influenced by the histological elements of the part in which the epigenesis occurs. If the influence of the part predominates, the newly formed material resembles the pre-existing normal tissue—and thus in morbid hypertrophy, in regeneration of lost parts, &c., the process is just the same as in ordinary nutrition. This important law, which plays a very active part in pathological epigenesis, I will for brevity term "the law of analogous formations."

NOTE.—Hypertrophy. This term, especially in what are called practical books, is often confounded with "thickening." An hypertrophy is a physiological formation—thickening is a result of inflammation. The pathological formation is not an exact representation of the parent tissue, and its position is interstitial or otherwise abnormal, arresting in a greater or less degree the functions of the organ into or around which the blastema has been transuded. Hypertrophies, on the contrary, are, as a general rule, histologically and anatomically perfect, and the functional power of the organ increases in the ratio of its growth. The exceptions to this rule are found in "abnormal accumulations of normal elements," to which Vogel's term "morbid hypertrophy" ought to be confined. If the term thickening is considered too ordinary, some other should be employed that will express the entire change that has occurred in the affected part, and not confound it with that which is, certainly an intense, but at the same time purely normal and conservative process.

The consideration of this subject has a practical as well as theoretic importance, especially to the surgeon, in the treatment of wounds attended with loss of substance. The reparative power varies in the different tissues—bone, for instance, is repaired perfectly by a new growth. I doubt if the skin ever is; at least I have never been satisfied by any evidence that has come within my limited observation that this tissue has been repaired to any extent, even the most minute, that comes under treatment, after the manner of its original formation; the new product being, like the parent tissue, fully capable of performing all its functions. When loss of substance occurs in the skin, experience teaches us that, instead of the delicately organized papillæ, which give to the surface its peculiar characteristic, it is patched with connective tissue, forming a dense unsightly cicatrix, and in those cases where the skin has been restored to apparent perfection, its morbid sensibility under all those slight impressions which it is its office to resist, and its proneness to ulceration under ordinary pressure, convince me, without the aid of the microscope, that the new formation has not reached the physiological standard.

It is certainly a question of the first importance to the practitioner, whether his patient is to be disfigured and maimed by his *cure*, or have the part restored, in good degree, to its original form and usefulness—that is to say, whether the injury is to be repaired by growth or by thickening, by homologous or analogous elements.

It would form no very difficult task to show the comparative procreative powers which the tissues possess, nor would it be altogether profitless; for, revealing the causes which hinder or accelerate restoration, it would suggest indications for treatment. Surgery, however, is not my province. I wish only to direct attention to the necessity of connecting definite ideas with terms in common use.

We see in fœtal development and growth a general condition of the capillaries, well marked, for instance, in bone; which, if compared with that of adult life, as the standard, would be pronounced a state of hyperæmia.

This state of vascular excitement is revived locally in adults, under the influence of exalted functional activity; and as its products are physiological growths (hypertrophies), I shall call it physiological hyperæmia.

It constitutes with other conditions the law of homologous formations.

*Formula* 1*st.*

Law of Homologous Formations.

Blastema—Albumen.

*Conditions.*

1. Physiological hyperæmia.
2. A physiological blastema.
3. The parent tissues in a physiological state.
4. The supply (of blastema) not to exceed the power to organize it wholly into homologous histological elements.

The law of homologous formations is composed of that assemblage of conditions, under the influence of which the tissues produce elements like their own from the blood.

The blastema is the pabulum from which they are immediately produced.

The first step in the process of development is the organization of the blastema; that is, its conversion into cells, by which, probably, appropriate inorganic materials are elaborated; or the blastema is fibrillated for the new growth, according, perhaps, as special materials are, or are not, required, as for instance, phosphate of lime, in the bones.

The second step is the development of these primitive forms into histological elements, identical in every respect (chemical and morphological identity) with those of the parent tissue.

Doubtless Mr. Huxley will yet bring to light all the hidden details of the metamorphosis of the blastema.

To this elaboration and development the reaction of the healthy parent tissue is essential. It is also essential that the supply of material shall not exceed the demand of the part, or its power to organize it wholly. This requisition is determined mainly by the degree of functional activity manifested.

The second and third conditions are obviously essential.

This law is fundamental. In the adult it is a *latent* force, that springs into action for good or evil, according as the exciting cause is physiological or pathological, that is to say, either exalted functional activity, or any cause that induces inflammatory action.

There can be no doubt that from its modified conditions, the great majority of pathological forms arise, and that all are influenced by it. For example, substitute pathological for physiological hyperæmia, and we have at once all the conditions broken but the second.

This state (pathological hyperæmia) constitutes the essential feature of inflammation. It may be excited by prolonged and excessive functional action, as well as by violence, or the ordinary causes of disease. The formations resulting from it are similar to those which flow from physiological hyperæmia, but not identical. They are analogous.

Hence we have the law (Vogel's) controlling these formations.

*Formula 2d.*

Law of Analogous Formations.

Blastema—Albumen.

*Conditions.*

1. Pathological hyperæmia.
2. The blastema physiologically organizable.

The departure of the parent tissue from the normal standard, and the excess of blastema, are included in the first condition.

These formations vary in their structural and chemical characters. Some can scarcely be distinguished from true growths (hypertrophies), or from the parent tissues; others form with the pre-existing tissues an amorphous mass of condensed matter, that can scarcely be resolved by the microscope. The conditions are altered in a corresponding degree; here we see the force of Vogel's remark, which has twice already enriched these pages, which ought to be emblazoned over the cases of every pathological cabinet.

"In proportion as the physiological properties of the parent tissue deviate from the normal type, so much the more heterogeneous will be the epigeneses." Analogy degenerates into heterogeneousness, the least perfect specimens forming the cancroid diseases of recent pathology.

In heterogeneous formations the same gradations may be observed. In cancroid diseases analogous elements abound. As we descend the scale, secondary development of the blastema fails; and finally, in the lowest forms, primary development is more or less imperfect, as seen in colloid cancer.

It has been stated, that to the elaboration and development of the blastema, the reaction of the healthy parent tissue is essential. This is, of course, diminished as consolidation takes place, by the interposition of analogous elements, until it almost ceases; perhaps, and most probably, from the interruption of nervous communication by the pressure of the analogous elements. Thus the blastema is rendered almost unorganizable, and absolutely incapable of being developed into either homologous or analogous formations. Heterogeneous products are alone evolved. So then, we may say there is a law controlling the formation of cancer.

*Formula 3d.*

Law of Heterogeneous Formations.

Blastema-Albumen.

*Condition.*

The parent tissue incapable of physiological reaction.

That this condition renders the blastema incapable of being developed into any homologous or analogous tissue, when absolute, may be demonstrated, and at the same time it can be shown that these two conditions: 1st, capacity in the blastema for development, and 2d, the reaction of the parent tissue, are not only essential to organization, but that they are distinct forces, though mutually dependent; thus, if an organizable blastema be received in a glass cup, no chemical process can convert it into bone or muscle; if received into a necrosed bone, it will not become organized; if received into a condensed tissue, it may be developed into a morbid product, but if it be received into a healthy tissue, it will be converted into homologous elements, under the law of homologous formations.

On the other hand, an unorganizable blastema cannot, under any condition of the parent tissue, be developed; it will either be absorbed or encysted, or it will decompose and be cast out. It therefore follows of necessity, that there is a force in the blastema as well as in the tissues; but it is equally evident that the nature of the blastema depends upon the condition of the tissue into which it is received. It is elaborated there.

Now as a pure albuminous solution cannot be developed of itself, we arrive at the conclusion, that elaboration consists in the proper combination of earthy and animal principles, and development in the formation and growth of histological elements from this nidus.

I have endeavored to trace in outline the course of the formative process, from the production of homologous elements to that of cancer-cells. I trust that I have shown that it is not essential that an hereditary diathesis should exist, or that any constitutional proclivity should usher in these formidable messengers of death. Such antecedents are favorable to their production, inasmuch as they affect the blastema, and they are the usual precursors of cancer; but this result may follow a bruise in a perfectly healthy man, and manifest itself as a primary local affection, or become constitutional and secondary.

Mr. Paget does not unite with those who hold this opinion; yet he admits that change of structure has some agency in the production of this disease, but has not shown how it is effected. If not by modifying the physiological conditions of the tissues in the manner indicated by Vogel, I would with deference ask, What is the mode? Should proof be required that the position assumed is based on true ground, it may be found in the analysis of Mr. Paget's description of the three great divisions of cancer, viz.: Scirrhus, Encephaloid, and Colloid, and by showing the degeneration of one form into the other.

Mr. Paget makes the following distinction between "innocent and malignant" tumors.

The chief distinctions are to be traced in certain characters which, in the malignant tumors or cancers, are superadded to those cited as belonging to the whole class (vol. 2, p. 10); that is, to "innocent tumors," which are "really imitations, so far as their structure is concerned, of the natural parts" (p. 11).

In other words, an innocent tumor is composed of analogous elements; to render this malignant something must be superadded. It only remains to detect, if possible, the origin of the foreign element and the causes of its malignancy.

Scirrhous or hard Cancer—Anatomy (Lecture 10). "The diseased mass is composed of the glandular and other tissues forming the mamma. These tissues are infiltrated by a substance that diminishes the size of the organ by its contraction, often to less than one-half its normal dimensions, thus altering the form of the breast, causing it to assume a lobular appearance, and drawing inwards towards itself the adjacent tissues." Microscopic Anatomy. The organ thus consolidated is further infiltrated by "a substance composed of two distinct parts. 1st. A fluid, solid, or nearly homogeneous substance, in which lie imbedded 2d. Certain cells, and other corpuscles. The intercellular substance very rarely assumes an appearance of fibrous texture; the cells are nucleated and multiform. This multiformity is a prominent feature of malignancy. I know no innocent tumor, except the cartilaginous, in which it is imitated. They resemble secreting gland cells."

This is essentially Mr. Paget's description of his "Typical Cancer." It is, evidently, *cirrhosis*, the analogous elements of which it is composed, having consolidated the organ, has crippled the formative forces, and rendered the part incapable of producing a higher organization than cells incapable of secondary development. Like secreting gland-cells, they perish and are reproduced.

Mr. Paget gives this "typical" cancer a middle place in a series, at the opposite ends of which are "acute" and "chronic" cancers.

"Acute cancers are rapid in progress, scarcely to be called hard; at the most, firm, tense, and elastic, even presenting a deceptive feeling of fluctuation. They are succulent, and suffused with vascularity, especially about their borders. The quantity of cancer-structure in them is very large, in proportion to the quantity of gland in which it has its seat; hence the section of an acute cancer appears more homogeneous, and the swelling is greater than in the typical form, the morbid material expanding the tissues involved. The surrounding tissues, also, are less closely connected with the cancer, which may appear as a distinct isolated tumor, rather than as an infiltration."

"In all these conditions the acute scirrhous cancers approximate to the characters of medullary cancers, and perhaps they are examples of an intermediate form of the disease, as seen in their more rapid growth, in the age of those in whom they usually occur (the young), and in their vascularity."

This is a description of encephaloid cancer, which I take it is intermediate between scirrhous and colloid cancers. There is no distinct account of the origin of the disease, or of the condition of the adjacent tissues. The tumor, however, is described as being suffused with vascularity at its borders. We may, therefore, reasonably suppose, that in the forming stage of that portion which has passed into encephaloid by the infiltration of cancer-cells into the thickened parent tissues, "vascularity" had existed, which produced the thickening and rendered the part incapable of physiological reaction. Cancer-cells, instead of analogous products, were then developed, and absorption removed the parent tissues and their analogous additions. So we may infer that the borders suffused with vascularity will, in their turn, become consolidated, and finally, infiltrated with cancer-cells.

"Chronic cancers, small, continuously becoming smaller, hard, knotted, and dry, adjacent tissues adherent, section concave, and shows more of the increased and indurated fibrous tissue of the breast than of the proper cancer-structure. All the history of chronic cancer accords with these signs of inactivity; they occur in those beyond the mean age; there is no increase of vascularity, or it is ruddy at the very seat of adhesion with the skin; the proper tissues suffer atrophy," &c.

What is meant by "increase and indurations," and by "adhesion?" If all this means inactivity and anæmia, I am unacquainted with the results of activity and hyperæmia.

It only remains to glance at colloid cancer.

"Colloid cancer. The colloid substance is clear and structureless, and sprinkled with minute dots, like oily and fatty molecules, which Dr. Jenner thinks are granules of phosphate of lime, and which to the naked eye may give it a peculiar milky or ochrey aspect, and sometimes it is beset with clusters of such molecules, resulting apparently from the degeneration of imbedded nuclei or imperfect cells," &c. &c.

"Lebert has published an exact analysis of this colloid matter, by Wurtz. The main results are, that it is quite unlike any variety of gelatin, being insoluble in water and containing only seven per cent. of nitrogen, a peculiarity which distinguishes it as well from all protein compounds, and from the materials of which (imperfectly and impurely as they have been examined) the essential structures of other cancers have been composed."

"Imbedded in the colloid substances, but in very uncertain quantity, are corpuscles of peculiar form; they are chiefly these nucleated cells, lie free in the colloid substance, or inclosed within large brood-cells, or grouped like an epithelium on the boundaries of the alveoli or cysts. These, the so-called colloid corpuscles, are small, granular, moderately transparent cells, of irregular shape, with small nuclei, or none."

"These are probably cancer-cells *hindered* or *modified* in their development by the peculiar circumstances of their formation, for with such as these, *more perfect cancer-cells* are sometimes found.

"If we look not to its structure alone, but as well to its clinical history, we shall find in it (colloid) all the distinctive features of the cancers.

"1. Its seats of election are those in which the medullary cancers are apt to occur.

"2. Like typical cancers, colloid infiltrates and replaces the natural tissues.

"3. Like them it repeats itself in other parts near to or distant from its primary seat.

"4. Colloid is often associated with other forms of cancer in the same mass.

"5. It recurs after removal.

"6. It may be derived hereditarily from parents having scirrhous cancer, or a parent having colloid may have offspring with medullary cancer."

Comment is needless.

Such are what I suppose to be the facts in the case. If this be admitted, the argument is a very simple one, and may be briefly stated.

There is but one law controlling the formation of histological elements, the conditions of which, when modified by any agent, give rise to morbid products. This is usually effected by increasing the quantity of the blastema, and by altering its constitution. Quantity and quality determine the nature of the formations, from homologous elements to ichor.

There is but one law and one blastema, and though the latter varies in its constitution to form the pabulum of the different tissues, this variation is a result of elaboration, and is a part of the process of organization.

There are three questions in opposition apparently to the foregoing views:

1. If it be true that cancer results in any case from inflammation, and that its formation is controlled by absolute laws, why not in every case?

There are no exceptions; so long as we remain in ignorance of the true nature and modes of action of the nervous fluid,(?) the primary morbid action of disease will be an occult remote cause, that will continue to be indicated, as such, under the vague terms "diathesis" and "proclivity." Take in illustration one of those cases of cancer which are of obscure origin. A tumor of small size is found in the breast, that had not been observed before. It is now incessantly handled, blistered, and anointed with irritating oils, until it ulcerates; it is then cauterized, to "change the action," which, only aggravating the evil, the tumor is pronounced a cancer, and the whole mystery is cleared up satisfactorily by the word diathesis, signifying thereby a pathological state of the blood.

I prefer to believe, until it is disproved, that the nervous fluid is electric fluid, and that a nerve in a state of exalted functional activity from a disturbance of the equilibrium of this subtle agent, is as reasonable an hypothesis as that the whole mass of the blood should be abnormal, to produce this little ganglion in the breast, and give no other evidence of it whatever.

2. Why does not every chronic inflammation terminate in cancer?

The pus-globule is a product of the formative process as well as the cancer-cell, and it is quite as heterogeneous; but being incapable of further development, and having laws peculiar to itself, relating to its office or function of disposing of the blastema when in excess, it acts as a conservative agent, and suggests the mode of treatment in cases that have resisted other measures for arresting inflammation.

3. Is there any constitutional cause for the disease?

Unquestionably. Still a local exciting cause will be required to render it effective, and the development of the blastema will be controlled by the conditions above-named. There may be degrees of capacity for development in the blastema, independent of the state of the tissues; but I cannot understand how this can be a local and not a general condition.

But I can understand a diathesis, the laws of which must be looked for in the nervous system; and I hope the distinguished author of the "Conditions and Course of the Life of Man" will, before long, make us familiar with the statutes.

[Part of this paper was addressed as a letter to my friend Dr. Carnochan: this will account for the frequent use of the pronoun I, and for the interrupted style of the article.]

